# The Discovery of Novel Selective D1 Dopaminergic Agonists: A-68930, A-77636, A-86929, and ABT-413

**DOI:** 10.1155/2011/424535

**Published:** 2011-03-24

**Authors:** Yvonne Connolly Martin

**Affiliations:** Department of Global Pharmaceutical Research and Development, Martin Consulting, Waukegan, IL 60064, USA

## Abstract

The novel selective D1 dopaminergic full agonists A-68930, A-77636 were discovered by the synthesis of molecules to probe the bioactive conformation of the partial agonist SKF-38393, by the use of this information to add D1 affinity and selectivity to a screening hit, and by traditional medicinal chemistry exploration of structure-activity relationships. The subsequent design of A-86929 and ABT-413 capitalized on these results, recently disclosed agonists, and traditional medicinal chemistry.

## 1. Introduction

For many years dopamine was considered to be biologically inert and acted merely as a precursor to the important neurotransmitters norepinephrine and dopamine [1]. However, Abbott's research on the biological properties of dopamine started in the late 1950s after the discovery of the monoamine oxidase inhibitor pargyline [2–4]. Using this compound Dr. Guy Everett showed that dopamine is an important neurotransmitter—a fact that was not universally accepted in the early 1960s [5–8]. He described dopamine as “the Cinderella compound,” unappreciated but not to be overlooked [1]. Although the role of dopamine in Parkinsonism was understood at this time, this was not an area of active investigation at Abbott Laboratories. Instead, monoamine oxidase inhibitors were targeted to the treatment of depression.

Somewhat later we had a small effort to discover an inhibitor of dopamine-*β*-hydroxylase, the enzyme that converts dopamine to norepinephrine [9]. Because norepinephrine raises blood pressure but dopamine does not, such compounds would be potential antihypertensives [10]. Although we had a robust QSAR, the project was abandoned before we found a novel inhibitor. 

A few years later Abbott had a joint project with Dr. Leon Goldberg from the University of Chicago. The objective was to design prodrugs of dopamine that would be selectively released in the kidney. Such agents would be useful in congestive heart failure and shock because they would not have the hemodynamic side effects of parenterally administered dopamine. They might be useful, at higher doses, as hypertensive agents [11, 12]. Our first prodrugs were alpha-amino acid amides of the amino group of dopamine. Although these prodrugs release dopamine in the kidney, the primary site of hydrolysis is the gut. Hence, they are not renalspecific [13, 14]. However, after a literature search we proposed the *γ*-glutamyl derivative. It is indeed released primarily in the kidney [15]. The compound was patented, but it was not developed further [16, 17]. The structure-activity relationships of these compounds and the failure to detect a useful QSAR are reviewed elsewhere [9].

Subsequent catechol amine research at Abbott focused on various adrenergic receptors with a goal to reduce dopaminergic effects. In support of this effort we developed 3D pharmacophore maps and 3D QSAR relationships for *α*_1_ and *α*_2_ adrenergic agonists and D2 dopaminergic agonists [18–20]. Previous publications summarize aspects of this research, particularly the 3D QSAR investigations [9, 21–23].

## 2. The Search for Selective D1 Agonists

This review emphasizes the computational chemistry support in the search at Abbott Laboratories for a full D1 dopaminergic agonist and summarizes the biological properties of the compounds. The project was started in 1986 with the appointment of Dr. John Kebabian, known for recognizing that there are at least two dopamine receptors [24], to lead an anti-Parkinsonism effort. He realized that because there was no selective D1 dopaminergic full agonist available for pharmacological and clinical testing, it was not known if targeting D1 receptors would have an advantage in treating Parkinsonism [25]. In the effort described below, Dr. Robert Schoenleber led the medicinal chemistry effort and Dr. Robert MacKenzie the biochemistry effort. The p*K*_i_ values for the D1 receptor were measured in homogenized rat caudate by their competition for binding of the selective antagonist [^125^I]SCH23982. Patricia Pavlik Hutchins contributed to the computational chemistry effort.

In the first phase of the project, a senior medicinal chemist from the adrenergic project selected catechol amines from the Abbott compound collection that he thought would have dopaminergic activity. Compound **1** (p*K*_i_ = 5.90) was discovered in this effort. However, this compound was also a full *α*_2_ adrenergic agonist, which would complicate assignment of its biological properties to D1 agonism.

This early biological testing also revealed that the phenyl group of the partial D1 agonist SKF-38393, **2**, increased the affinity for the D1 receptor by nearly 100-fold (p*K*_i_ = 7.9) compared to the desphenyl analogue **3** (p*K*_i_ = 5.0). Simultaneously, it abolished affinity for the D2 dopaminergic receptor [26]. Could we locate the 3D space that the phenyl group occupies with respect to the required basic nitrogen and phenolic OH group? We attacked this problem with a close collaboration between synthesis and 3D modeling [21]. 

**Figure 2:**
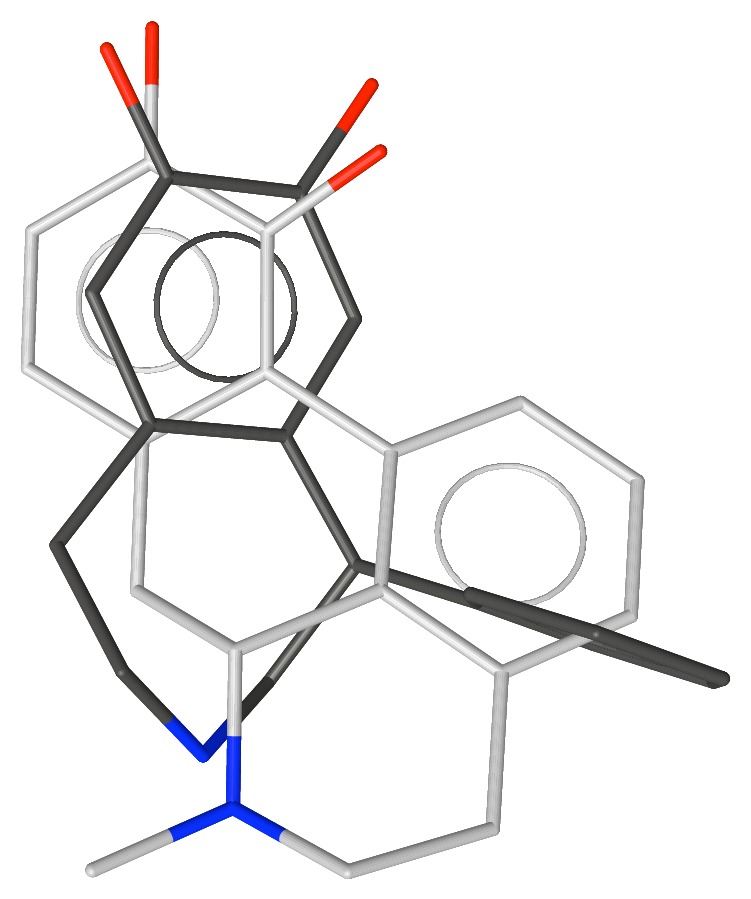
Apomorphine superimposed over the conformation of SKF-38393 with the phenyl group equatorial.

**Figure 3:**
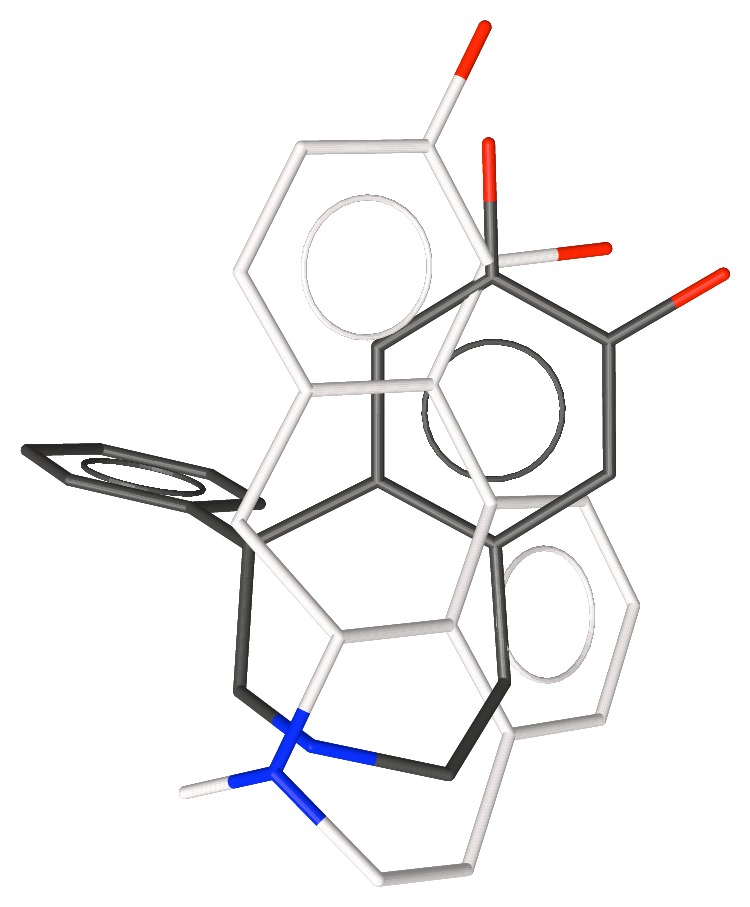
Apomorphine superimposed over the conformation of SKF-38393 with the phenyl group axial.

We explored the conformations of SKF-38393 by generating conformations with distance geometry [27] and minimizing them with MMP2 [28]. This revealed two conformations, one with the phenyl group equatorial and a second with it axial. MMP2 suggests that the equatorial conformer is slightly more stable, but AM1 [29] favors the axial conformation—neither difference was greater than 0.5 kcal/mole. These conformations are shown in Figures 2 and 3 aligned with apomorphine **4**, a nonselective D1/D2 agonist (D1 p*K*_i_ = 6.4). Note that because the bioactive stereoisomer of each compound is known, the alignments as shown overlap shapes as well as pharmacophore features.

Note the better alignment of the equatorial form of SKF-38393 with apomorphine. Although the noncatechol aromatic ring of apomorphine increases affinity for the D1 receptor approximately 100-fold, this compound is still a potent D2 agonist. Hence, it was not clear if the noncatechol aromatic ring is in the selectivity pocket or not. Note also that SKF-38393 protrudes farther to the right in the figure than does apomorphine.

**Figure 4:**
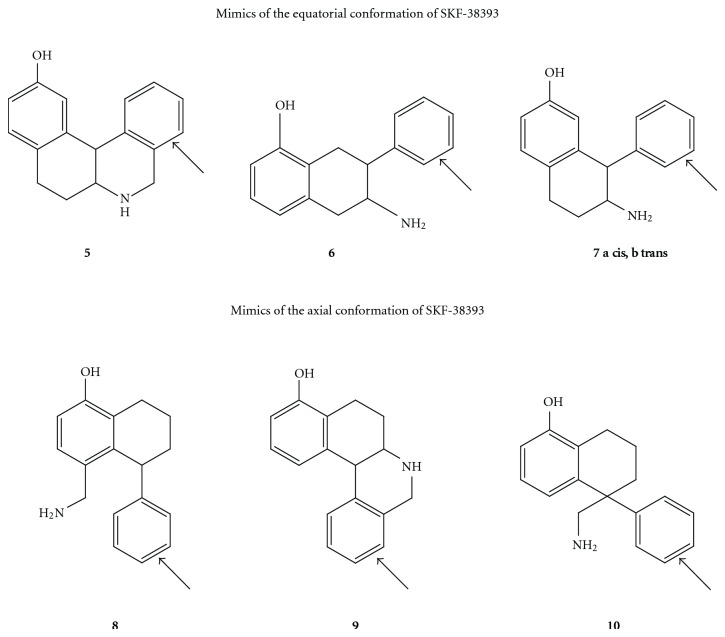
The structures of the compounds synthesized to probe the bioactive conformation of SKF-38393. Pairs of molecules were made with and without the phenyl group indicated by the arrow.

However, both superpositions look reasonable. Because molecular modeling could not answer the question of the bioactive conformation of SKF-38393, it was necessary to synthesize molecules to probe the location of the D1 accessory phenyl binding site. Molecular modeling and synthetic feasibility guided the design of seven pairs of molecules, each with and without the potential phenyl binding group. Structures **5**–**10** in Figure 4 show the phenyl-substituted compounds [21, 30, 31]. 

**Figure 5:**
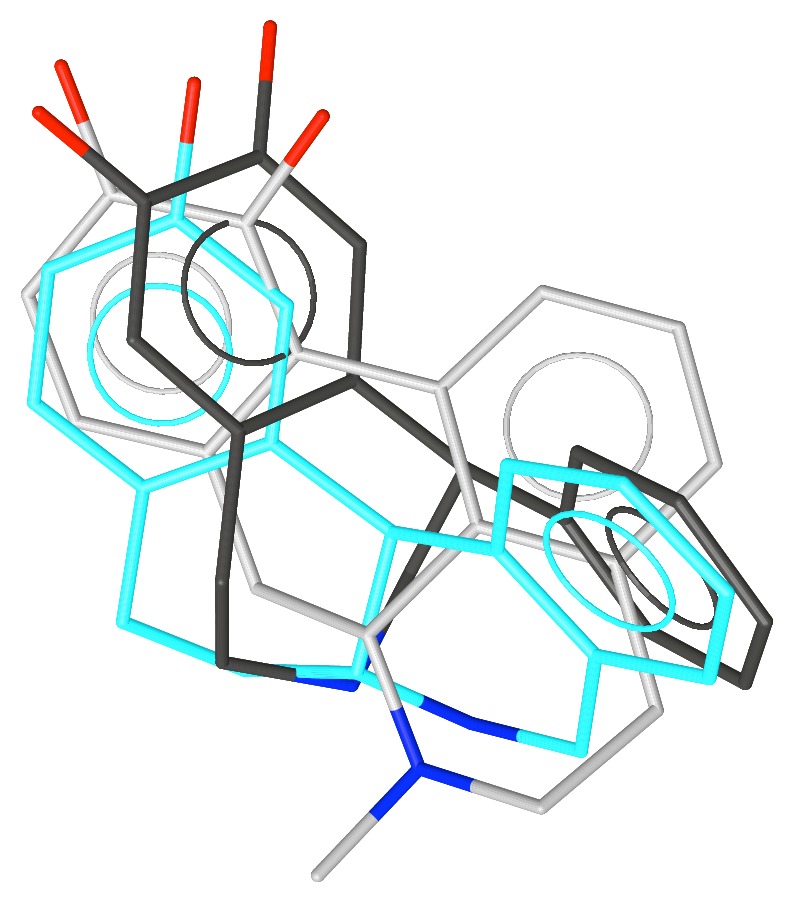
The superposition of apomorphine, the equatorial conformation of SKF-38393, and Compound **5**, which shows the “phenyl boost”.

**Figure 6:**
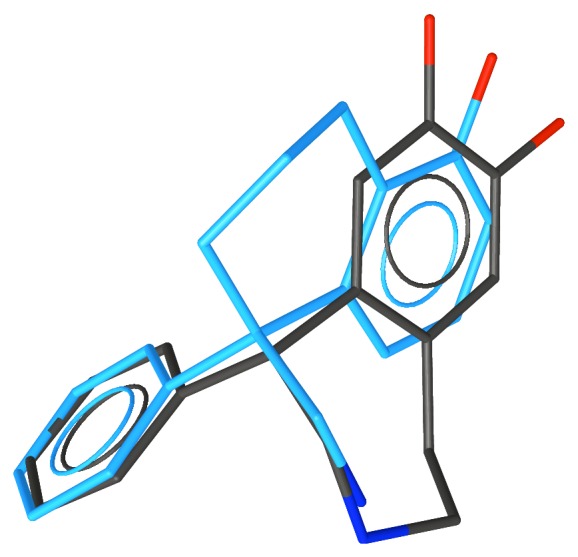
The superposition of apomorphine, the axial conformation of SKF-38393, and Compound **10**, which does not show the “phenyl boost”.

Although none of the synthesized compounds was very potent, biological testing revealed that increased affinity in the phenyl analogue was seen for only those compounds for which the added phenyl group is in the equatorial conformation. Hence, the bioactive conformation of SKF-38393 has the pendant phenyl group equatorial.  Figure 5 shows an example of one of the compounds that shows increased affinity due to the added phenyl group and Figure 6 shows an example of one of the compounds that does not show such an increase in affinity.

**Figure 7:**
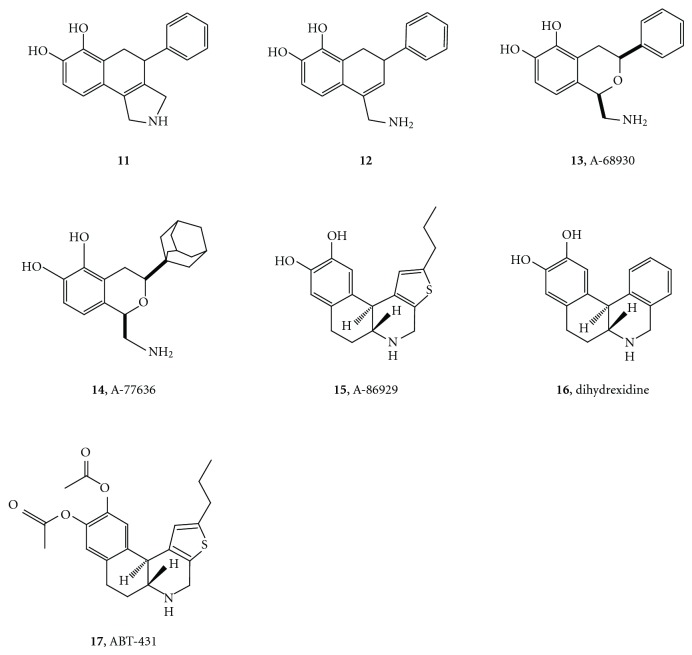
The structures of compounds **11**–**17** synthesized to further probe the accessory binding site on the D1 receptor.

**Figure 8:**
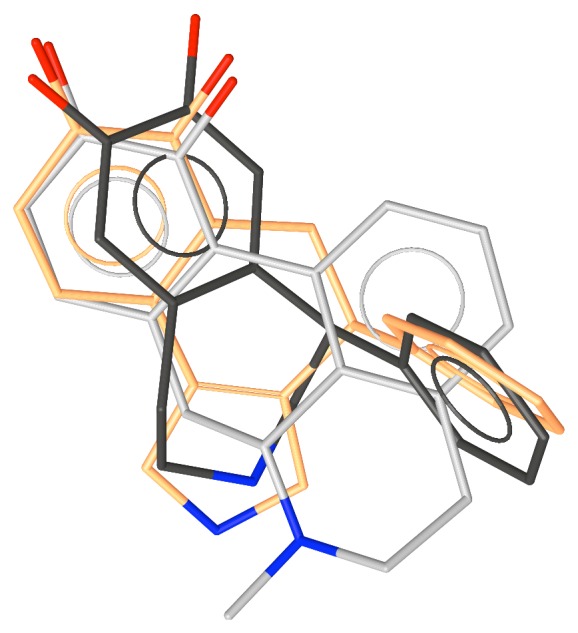
The superposition of apomorphine, the equatorial conformation of SKF-38393 and Compound **11**.

The new information provided the clue as to how to transform **1** (p*K*_i_ = 5.9) into a D1 selective compound, **11**, Figures 7 and 8. As predicted, **11** is a potent full agonist (p*K*_i_ = 7.2). Although traditional medicinal chemistry would have suggested compound **12** for synthesis, in fact the desphenyl analogue in our collection was identified by 3D searching with Aladdin [32]. 

**Figure 9:**
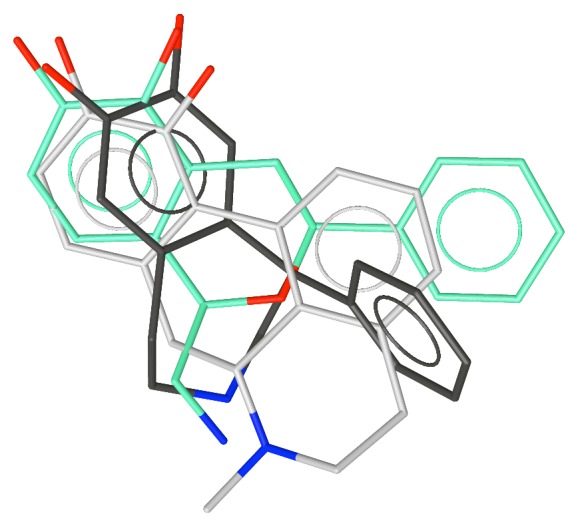
The superposition of the apomorphine, the equatorial conformation of SKF-38393 and A-68930.

Traditional medicinal chemistry followup of **12** resulted in the isochroman series, **13** (phenyl analogue A-68930, p*K*_i_ = 8.5), shown in Figure 9. The basis of the enhanced affinity of A-68930 compared to **12** is not clear—it could be due to the increased flexibility of the heterocycle ring or to some electronic effect of the oxygen, perhaps on the basicity of the amino group.

Drs. Michael DeNinno and Michael Michaelides led the effort to explore the structure-activity relationships of the isochromans [33–38]. Although the *trans*-isomer is more stable, the synthesis provided the more potent *cis*-isomers. The structure-activity data revealed that what we had called the “phenyl binding site” could accommodate such substituents as 1-adamantyl. Substitution of the catechol 6-OH group by Br, H, or OMe results in compounds that are antagonists in vitro but agonists in the 6-hydroxydopamine rotation test [37].

A study of A-68930 was performed in rats bearing a unilateral 6-hydroxydopamine lesion of the neostriatal neurons showed a greater than 20-hour duration of contralateral turning [39]. However, on the second day of treatment, essentially no response was seen [40]. Although we had feared that the catechol would be susceptible to rapid metabolic deactivation, instead the effects of the compound are very long acting.

The 1-adamantyl analogue A-77636, structure **14**, is also a potent agonist (p*K*_i_ = 7.4). It, too, is active in the 6-hydroxydopamine model for more than 20 hours [38]. Additionally, it increases locomotor activity and decreases the Parkinsonism-like symptoms in MPTP-treated marmosets after either oral or subcutaneous administration. The enantiomer with lower affinity for the D1 receptor does not show these pharmacological effects. The compound also showed anti-Parkinsonism activity in monkeys and appeared to have a favorable profile with respect to dyskinesia [41]. However, it shows, too, diminished activity on the second day of administration. 

As the project accumulated structure-activity data, we developed a 3D QSAR model to be used to forecast affinity of proposed compounds [22]. Although this was an attractive model, while the observed p*K*_i_s for the set of 46 isochromans varied from 4.0 to 9.28 with a standard deviation of 1.06 logs, the predicted p*K*_i_s for compounds not included in the model varied from 5.4 to 7.8 with a standard deviation of 0.52 logs. Thus the QSAR model overpredicted the affinity of low-affinity compounds and underpredicted the affinity of high-affinity compounds.

**Figure 10:**
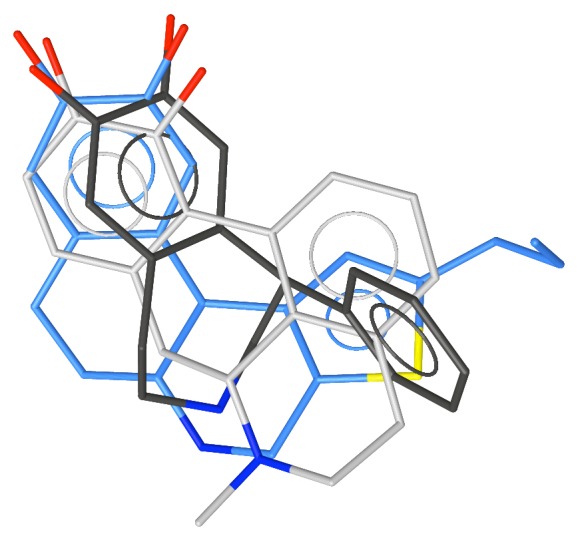
The superposition of apomorphine, the equatorial conformation of SKF-38393, and **15** (A-86929).

**Figure 11:**
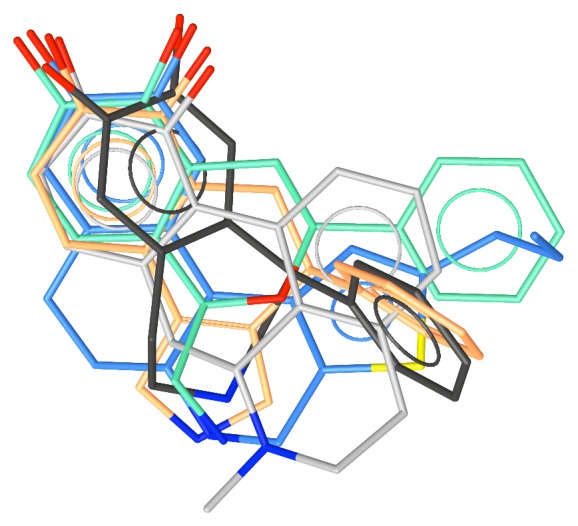
The superposition of the various D1 agonists considered in this report.

Nevertheless, this 3D QSAR model for D1 affinity was combined with our QSARs for D2 and *α*_2_ agonists to evaluate proposed alternative series to the isochromans. In support of this effort, we generated all of the low-energy conformers of the stereoisomers of approximately 100 proposed compounds and used them to provide a forecast of the D1, D2, and *α*_2_ affinities of the compounds. The most potent of these compounds is A-86929 **15** (p*K*_i_ = 7.3), Figure 10 [42]. It was designed from the knowledge of the 3D structure-activity relationships of the compounds synthesized to locate the phenyl-binding pocket and of the isochromans as well as the recently disclosed agonist, dexedrine, **16** [43].  Figure 11 shows the superposition of the various potent D1 agonists.

After Drs. Kebabian and Schoenleber left Abbott Laboratories, further exploration of the D1 agonists was led by Dr. Kazumi Shiosaki.

In contrast to the isochroman analogues, A-86929 has a short duration of action and it maintains efficacy in the rat rotation model upon repeated subcutaneous administration. The O-diacetyl pro-drug ABT-431 (adrogolide), **17**, shows a similar pharmacological profile, but it is more stable in the solid state [42]. A-86929 is also active in the MPTP marmoset model of Parkinsonism [44]. In a complex marmoset model of Parkinsonism, it produced a more naturalistic response than L-dopa [44]. There is also a greater than tenfold separation between the dose in rats that produces contralateral rotation in the 6-hydroxydopamine-lesioned rat and that which produces seizures. On the basis of this favorable profile, ABT-431 was tested in human Parkinsonism patients. It showed efficacy equivalent to that of L-DOPA, with the same tendency to induce dyskinesia as L-DOPA [45, 46]. In man ABT has a low oral bioavailability (±4%) due to a high hepatic “first-pass” metabolism [47]. This limitation has been circumvented by oral inhalation formulations for intrapulmonary delivery that greatly increase its bioavailability [48]. Because ABT-431 showed no advantage in decreased dyskinesia compared to L-DOPA and because of the challenge of finding an oral dose formulation, it was not further developed.

ABT-431 remains an important pharmacological tool to probe the role of D1 agonism in various physiological and psychological properties. For example, it was shown that ABT-431 can reduce the ability of cocaine to induce cocaine-seeking behavior and does not itself induce cocaine-seeking behavior in a rodent model of cocaine craving and relapse [49]. In human cocaine abusers, intravenous ABT-431 reduces cocaine craving and other cocaine-induced subjective effects [50]. Animal abuse liability studies indicate that it is unlikely to have abuse potential in man. ABT-431 has also been reported to reverse haloperidol-induced cognitive deficits in monkeys, suggesting that it may be an effective treatment for the cognitive dysfunction associated with aging and disease [47]. A short-term administration of ABT-431 also reverses for more than a year the impairments to working memory produced by chronic blockade of dopamine D2 receptors by antipsychotic drugs [51]. Furthermore, an intermittent, sensitizing regimen of ABT-431 dramatically enhances working memory performance in aged rhesus monkeys, an effect that was still evident for >1 year after cessation of D1 treatment [52]. 

## 3. Conclusion

These studies show the results of the synergism between molecular modeling, chemical synthesis, biological screening of a database of potential molecules, 3D database searching, and traditional medicinal chemistry. The D1 selective agonists, although they are not superior to L-DOPA in the treatment of Parkinsonism, are useful probes for the role of D1 agonism in living organisms.

## Figures and Tables

**Figure 1:**
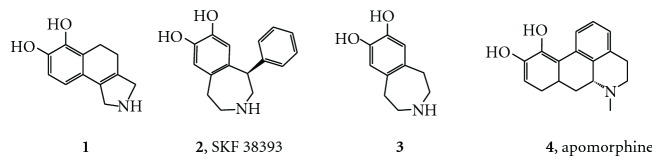
The structures of compounds **1**–**4**.
